# Notes and News

**Published:** 1893-01-14

**Authors:** 


### NOTES AND NEWS.

COLLECTED AND COMMUNICATED.

AN English edition is now published of "La Semaine Medicale," entitled "The Medical Week." It is a record of important medical cases from the chief scientific centres of the globe, and, therefore, from the diversity of experience, is likely to prove interesting to many. It can be obtained at 13, Rue de l'Abbé de l'Epée, Paris.

A USEFUL little society exists in France, which charges itself with pensioning members of the medical profession who fall victims to their duty. In case of death, the relatives receive the benefaction. The widow of Dr. Piasecki, of Havre, who fell a victim to his devotion to duty, is the latest recipient of the bounty of this institution.

THE will of the late Rev. James Spurrell provides that the munificent sum of £181,500 shall be divided amongst the charities. The medical charities remembered are the London Hospital, the Brompton Hospital for Consumption, £4,000; the Hospital for Incurables, £4,000; the Brighton and Hove Dispensary, £2,000; and the Brighton Hospital for Sick Children. As neither relations nor friends of the testator have been forgotten, this much-needed help to our charities will be welcomed by all.

A MEETING was held on December 30th, at the offices of the Hospital Saturday Fund in Farringdon Road, in furtherance of the proposed athletic and cycling sports in aid of the hospitals of the metropolis. The chief business was the election of a council, from which a practicable committee will be chosen to carry out the idea. There was a large number of prominent athletes present. It was stated by a member of the Stanley Bicycle Club that that club would offer a valuable trophy to be competed for in connection with the charity festival, probably for a five miles scratch race. A hope that other clubs would also give prizes was expressed. A meeting of the newly-elected council was afterwards held, but little was done beyond fixing the date for the next meeting.

IT is interesting to learn that statistics show a decided decrease in insanity in the United States. In 1880 the ratio of insane to sane was 1 to 615. During the next decade the numbers were 1 to 620.

AN addition has been recently made in the periodical literature for girls by the publication of *The Young Gentlewoman*. This magazine is to appear monthly, is largely illustrated, and the price sixpence. Whilst possessing many excellent qualities which will render it popular, *The Young Gentlewoman*, to our mind, possesses some grave faults. Foremost of these is the fostering of that tendency towards notoriety which is a besetting weakness of the age. We are surprised that parents have not more common sense than to permit "Lizzie," aged nine, and "Algie," aged ten, to figure as public characters pourtrayed in print as heroes of no very wonderful performances. Thus thrust forward into notice, it would not be surprising if Lizzie and Algie were to become very unpleasant little prigs, their claims to distinctiion ending as they began in a first and last appearance in *The Young Gentlewoman*. It is not a pleasant indication of the "tone" of the present day that this periodical should find it a profitable form of advertisement to thrust the young idea forward in this objectionable manner. The success of *The Young Gentlewoman* on the present lines will be both a reflection upon parents and teachers of the present age. Many of the articles contained in it are excellent, and we must hope that the proprietors will see their way to a consistently high tone throughout the magazine in the immediate future.

THE case against the Poplar Hospital for Accidents is a typical illustration of the hasty verdict that is passed against the medical charities in general, prior to an investigation. The Poplar Hospital is amongst the most useful of our charities, and indeed, so pressing are the calls upon it in that realm of accidents where it is situated, that the management have to be most watchful that the most urgent cases are those which shall not go away uncared for. This watchfulness caused the removal of the man Eve at a time when the nursing staff was short of hands and his a difficult case of mania added to injuries from burns. It would appear that the only fault lay in the want of consideration shown to his relatives by the minor officials of the hospital. The relatives themselves appear to have been equally inconsiderate of Eve himself, so that they could not rightly bring forward the charge they did. We trust that hospital officials will learn the lesson that great forbearance and consideration are essential qualities of their office, for in dealing with both patients and their friends such qualities are constantly called into requisition. In the case of the Poplar Hospital, we hope that the staff will avoid indiscretions of the kind charged to them in the future, and that the public will tender the money necessary to enable this most useful institution to more adequately meet the requirements of the neighbourhood. The hospital has been badly used in being forced to close one ward, and it should certainly not be allowed to complain of the want of a most necessary isolation ward any longer.

